# *Streptococcusthermophilus* ST285 Alters Pro-Inflammatory to Anti-Inflammatory Cytokine Secretion against Multiple Sclerosis Peptide in Mice

**DOI:** 10.3390/brainsci10020126

**Published:** 2020-02-23

**Authors:** Narges Dargahi, John Matsoukas, Vasso Apostolopoulos

**Affiliations:** 1 Institute for Health and Sport, Victoria University, Melbourne VIC 3030, Australia; narges.dargahi@vu.edu.au; 2 NewDrug, Patras Science Park, 26500 Patras, Greece; imats1953@gmail.com

**Keywords:** probiotics, *Streptococcus thermophilus*, ST285, MBP_83–99_ peptide, mannan, immune modulation, multiple sclerosis, agonist peptide

## Abstract

Probiotic bacteria have beneficial effects to the development and maintenance of a healthy microflora that subsequently has health benefits to humans. Some of the health benefits attributed to probiotics have been noted to be via their immune modulatory properties suppressing inflammatory conditions. Hence, probiotics have become prominent in recent years of investigation with regard to their health benefits. As such, in the current study, we determined the effects of *Streptococcus thermophilus* to agonist MBP_83–99_ peptide immunized mouse spleen cells. It was noted that *Streptococcus thermophilus* induced a significant increase in the expression of anti-inflammatory IL-4, IL-5, IL-10 cytokines, and decreased the secretion of pro-inflammatory IL-1β and IFN-γ. Regular consumption of *Streptococcus thermophilus* may therefore be beneficial in the management and treatment of autoimmune diseases such as multiple sclerosis.

## 1. Introduction

There is an increasing trend in immune-mediated disorders across the world that is believed to be in part, a result of intestinal dysbiosis. The imbalance in the intestinal ecosystem can lead to a dysfunctional immune system that consequently causes immune disorders including autoimmune diseases (multiple sclerosis, MS) and other inflammatory disorders [1,2]. Probiotics have long been implicated for the overall improvement of health and the management of a number of health conditions including infection, constipation, allergies, and autoimmune diseases, and are either consumed in the form of different fermented foods and dairy products or taken as capsules. In either case, there is strong evidence that suggests that the ingestion of probiotics can alter intestinal dysbiosis and relieve dysfunctionality complications, with subsequent improvements to health [3].

Probiotic bacteria have been evolved inside the human intestinal tract (GIT), and through this co-evolution, the gut and its microbiome have developed a symbiotic relationship that is of mutual benefit. While the GIT microflora relies on the gut’s warm habitat and food content, in return, it not only provides numerous unique bioactive components such as vitamins B and K, minerals, short chain fatty acids (SCF), and miosins to the host, but it also assists in modulating the immune system [3]. In fact, probiotics are able to modulate monocytes, macrophages, B cells, T helper (h)1, Th2, Th17, regulatory T cells (Treg), natural killer (NK) cells, and dendritic cells (DC) [3,4,5,6].

Chronic inflammation is the pathophysiological condition involved in neuro-degenerative disorders including MS, Parkinson’s disease, and Alzheimer’s disease [7,8]. There is cross-talk between the gut microbiota and the central nervous system (CNS) [8,9,10], known as the gut–brain axis. An insufficient or imbalanced GIT microflora can also lead to dysfunctions in the gut–brain axis and the pathogenesis of a number of diseases inside the GIT (such as inflammatory bowel disease, IBD) and outside the GIT (such as the CNS). Experimental autoimmune encephalomyelitis (EAE) is an animal model of human MS that has been used to study the effects of probiotic bacteria on CNS [11,12]. One of the safe and appropriate ways to modulate T cells in MS is to orally administer specific autoantigens [13,14]. Administration of Bifidobacteria or Lactobacteria probiotic strains to mice has been shown to increase Treg cells and tumor growth factor (TGF)-β levels and reduce the severity of EAE clinical symptoms, in parallel with improvement in the regeneration of myelin in the spinal cord compared to the control [15,16]. Administration of both Bifidobacteria and Lactobacteria strains induce an additional significant delay in the onset of EAE and related clinical symptoms, together with a substantial reduction of mononuclear infiltration into the CNS, and increased level of Treg cells of the CD4^+^CD25^+^Foxp3^+^ phenotype in mouse spleen and lymph nodes.

In SJL/J mice, immunization with the MBP_83–99_ peptide mixed with mycobacterium stimulates autoimmune CD4^+^ T cells in mice, and induces EAE [7,17]. Major histocompatibility complex (MHC) class II H-2^s^ haplotype in the SJL/J mouse strain resembles many clinical, histopathological, and immunological characteristics of human MS, thus the SJL/J mouse is regularly used for immunization studies. Different peptides are immunogenic in different mouse strains however, in the SJL/J mouse strain, the peptide MBP_81–100_ binds to MHC class II H-2^s^ with high affinity with the minimum epitope being MBP_83–99_ [7,17]. As such, the MBP_83–99_ epitope has been used as an agonist peptide to immunize mice for the activation of CD4^+^ T cells [7,17]. We have shown that injection of the MBP_83-99_ peptide conjugated to the carrier mannan or mixed in complete Freund’s adjuvant induces Th1 pro-inflammatory interferon-gamma (IFN γ-g) secreting CD4^+^ T cells [18,19,20,21,22,23,24,25]. Studies have shown that there is a cross-reactivity between the MBP self-peptide and some microbial peptides (i.e., UL15, PMM) for Hy.1B11 T cell receptor (TCR), which has been isolated from a patient with MS. It has been highlighted that there are chemical interactions underlying the recognition mechanisms between TCR and the peptides presented by MHC proteins, as a critical constituent in adaptive immune responses to foreign antigens [26].

The Streptococcus genus constitutes over 100 species, amongst which *S. thermophilus* (ST) are non-pathogenic and food related bacteria that represent outstanding technological features in the food industry [27]. ST are commonly used as secondary starter cultures in dairy products to transform lactose into lactic acid and to acidify the pH of milk [27,28], contributing to both the fermentation and flavoring of dairy products [29]. Most probiotics belong to lactic acid bacteria (LAB); Gram-positive lactic acid producing bacteria that include lactobacilli, bifidobacterial, and enterococci [3]. As such, live LABs are not only used in foods for their health benefits, but exopolysaccharide-producing strains of ST such as ST1342, ST1275, and ST285 are generally used due to their beneficial properties (i.e., relieving lactose intolerance and suppressing acute conditions such as acute ulcerative colitis) [29]. Additionally, experimental studies designed to investigate the effect of VSL3 (Streptococcus, Bifidobacterium, and Lactobacillus species) on the peripheral immune system and the GIT microbiota in MS patients and healthy subjects showed improved abundance of many taxa with enriched taxa mainly consisting of Lactobacillus, Streptococcus, and Bifidobacterium. VSL3 also induced peripheral anti-inflammatory immune responses [30].

We recently showed that ST bacteria have anti-inflammatory properties [29]. U937 pro-monocytic cell line co-cultured with three ST bacteria (ST1342, ST1275 and ST285) induced an anti-inflammatory profile [29]. ST285 was further shown to have immune modulating effects via gene arrays to human peripheral blood mononuclear cells (PBMC) [31] and monocyte cells isolated from PBMC [32]. Herein, we immunized SJL/J mice with agonist MBP_83–99_ peptide conjugated to mannan three times, isolated spleen cells, and after re-stimulation of spleen cells with the MBP_83–99_ peptide, IFN-γ was secreted by spleen cells. Re-stimulation of spleen cells with the MBP_83–99_ peptide in the presence of ST285 probiotics was able to downregulate IFN-γ responses and stimulate the Th2, IL-4, IL-5, and IL-10 cytokine profile. These studies show that probiotics are able to modulate and alter the immune profile of MBP_83–99_ specific cells to anti-inflammatory, which warrant in vivo EAE mouse experiments and hold promise as a therapeutic alternative approach to MS in human clinical trials.

## 2. Materials and Methods

### 2.1. Bacterial Strains

Pure bacterial cultures of *S. thermophilus* 285 (ST285) were obtained from the Victoria University culture collection (Werribee, Vic, Australia). Stock cultures were stored in cryobeads at −80 °C. Prior to each experiment, the cultures were propagated in M17 broth (Oxoid, Denmark) with 20 g/L lactose and incubated at 37 °C under aerobic conditions. Bacteria were also cultured on M17 agar (1.5% *w/v* agar) with 20 g/L lactose (Oxoid, Denmark) to assess the characteristics, morphology, purity, and Gram-positive confirmation [1].

### 2.2. Preparation of Live Bacterial Suspensions

Media were prepared and autoclaved at 121 °C for 15 min prior to the experiments. Bacterial cultures were grown three times in M17 broth with 20 g/L lactose at 37 °C aerobically for 18 h with a 1% inoculum transfer rate [33]. Cultures grow optimally at 37–42 °C for 24 h [29]. The growth period of cultures were consistent at 18 h (at the end of the exponential growth phase) and before the stationary growth phase to prevent cell lysis.

### 2.3. Enumeration of Bacterial Cells

For the actual experiment, bacteria were grown in broth media to the stationary phase at 37 °C aerobically, pelleted by centrifugation (6000× g) for 15 min at 4 °C, transferred, and resuspended in Dulbecco’s phosphate-buffered saline, pH 7.4 (Invitrogen, Pty Ltd. Australia). The bacterial density in suspension was adjusted to 10^8^ colony forming units (cfu)/mL for final concentration by determining the optical density at 600 nm, followed by two washes with Dulbecco’s phosphate-buffered saline. These samples constituted the live-cell suspensions and were resuspended in the Roswell Park Memorial Institute (RPMI) 1640 culture media prior to co-culturing with spleen cells [1].

### 2.4. Mouse Experimental Procedures

#### 2.4.1. Mice, Conjugates, and Immunization Schedule

Female SJL/J mice, aged 6–9 weeks, used in all experiments were purchased from the Animal Resources Center (ARC, Perth, Australia), and accommodated at the animal house (Victoria University, Werribee campus, Melbourne, Australia). All mice were ensured free access to water and food, and were housed in a temperature controlled room with a 12 h day 12 h night cycle. All immunizations were conducted according to the guidelines of the Australian Code of Practice for the Care and Use of Animals for Scientific Purposes and the study was approved by the Victoria University Animal Ethics Committee (AEC15/013) of Victoria University, Melbourne, Australia.

The MBP_83–99_ agonist peptide of over 99% purity with (KG)_5_ at the C-terminus was conjugated to mannan via a method previously described [34,35,36,37,38]. Briefly, 14 mg of mannan (Sigma, VIC Australia) was oxidized in sodium carbonate buffer and 0.1 M sodium periodate at 4 °C after which ethylene glycol was added and incubated for 30 min at 4 °C. Oxidized mannan comprising aldehyde groups was passed through a PD-10 column (Sigma, VIC Australia) pre-equilibrated in carbonate-bicarbonate buffer pH 9.0 and 2 mL of oxidized mannan was collected and 1 mg of MBP_83–99_-(KG)_5_ peptide was added and allowed to react overnight at room temperature in the dark. The resultant MBP_83–99_-(KG)_5_-mannan conjugate was used to immunize the mice.

The MBP_83–99_ mannan peptide conjugate (50 μg/mouse) was injected in the SJL/J mice subcutaneously into the base of the tail, three times, every two weeks [17]. This conjugate has been shown to induce T cell proliferation and IFN-γ cytokine secretion to the agonist MBP_83–99_ peptide in SJL/J mice [17,19,23,24]. Ten to fourteen days after the three injections, spleen cells were isolated, red blood cells were lysed using 0.73% NH_4_Cl, and counted.

#### 2.4.2. Isolation of Spleen Cells and In Vitro Stimulation with ST285

Spleen cells were resuspended in RPMI 1640 media supplemented with 10% heat-inactivated fetal bovine serum (Invitrogen, Pty Ltd. Australia), 1% antibiotic-antimycotic solution, and 2 mM L-glutamine in T75 cm^2^ cell culture flasks. Mouse spleen cells (1 × 10^7^) in RPMI media only was used as the negative control, 5 µg/mL recall agonist MBP_83–99_ peptide was used as the recall control, or 1 × 10^8^ ST285 bacteria were added together with the MBP_83–99_ peptide, and cultured at 37 °C, 5% CO_2_ for 24 h [29]. We previously showed that 24 h co-culture was adequate for the stimulation of the monocyte/macrophage cell line, human peripheral blood mononuclear cells, and human monocytes isolated from peripheral blood mononuclear cells [29,32]. At the end of the culture period, cells were transferred into falcon tubes, and centrifuged for 5 min at 1200 rpm to pellet the cells. All cell-free supernatants were collected and frozen at −20 °C until cytokine analysis.

### 2.5. Cytokine Production Analysis

Cytokine secretion of the spleen cell culture supernatants was analyzed by commercially available capture and detection antibodies in a Bioplex multiplex bead assay for a panel of nine mouse cytokines and chemokines using a 9-plex kit (BioRad, Melbourne Australia) to measure Interleukin (IL)-1β, IL-2, IL-4, IL-5, IL-6, IL-10, GM-CSF, TNF-α, and IFN-γ. Cell-free supernatants were collected and the assay procedures were performed according to the manufacturer’s instructions. Briefly, a flat bottom 96-well plate was coated with 1× coupled beads and washed twice, followed by adding the standard serial dilutions, blanks, and samples to assigned wells. Post incubation at shaking at room temperature, plates were washed twice, adequate 1× detection antibody was added, and incubated at room temperature. Plates were washed three times and 1× Streptavidin Phycoerythrin (SA-PE) stop solution was added to each well, followed by incubating at room temperature and washing. Data collection was repeated twice, data were expressed as the mean cytokine response minus background (pg/mL) of each treatment from three replicate wells, plus or minus the standard error of the mean.

### 2.6. Statistical Analysis

Significant differences between all treatment groups were tested by analysis of variance (ANOVA) using the Statistical Package for the Social Sciences for Windows 25.0 (SPSS; IBM Corp), followed by a comparison between treatments performed by Tukey’s honest significance test/degree and Fisher’s least significant difference method, with a level of significance defined as *p* < 0.05.

## 3. Results

### 3.1. ST285 Reduces Pro-Inflammatory TNF-α and IFN-γ Production by MBP_83–99_ Primed Mouse Splenocytes

**Figure 1:**
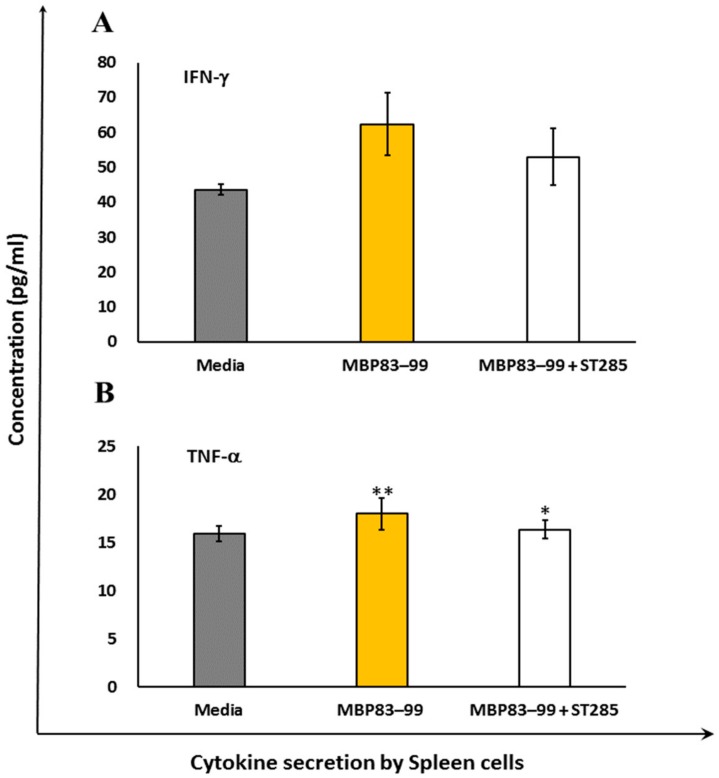
*S. thermophilus* 285 reduces pro-inflammatory cytokine production by mouse splenocytes. Spleen cells isolated from immunized mice (*n* = 3) were stimulated with *S. thermophilus* (ST) ST285 and recall agonist MBP_83–99_ peptide for 24 h and secretion of (**A**) IFN-γ and (**B**) TNF-α were measured. Recall MBP_83–99_ peptide was used as an internal positive control, and media refers to spleen cells from immunized mice (*n* = 3) without any additional recall peptide, or ST285 probiotic bacteria plus the MBP_83–99_ peptide. Means of two different readings of three replicate experiments were measured and analyzed. The means of readings for *n* = 3 mice were calculated and presented as plus or minus (±) the standard error of the mean. Symbols represent the *p* value for the Tukey test (one way ANOVA) where * *p* < 0.05 and ** *p* < 0.01.

Interferon gamma (IFN-γ) is a pro-inflammatory Th1 cytokine involved in macrophage activation and cellular immunity. IFN-γ promotes Th1 cells and inhibits Th2 anti-inflammatory cells. In MS, IFN-γ is induced following CD4^+^ T cell activation by agonist peptide MBP_83–99_. SJL/J mice immunized with MBP_83–99_–mannan conjugates induced IFN-γ responses by spleen cells, following overnight MBP_83–99_ peptide re-stimulation (Figure 1A, *p* < 0.01). Spleen cells re-stimulated with the agonist MBP_83–99_ peptide in the presence of ST285 reduced IFN-γ cytokine secretion (Figure 1A, *p* < 0.05). TNF-α, a Th1 cytokine, was not secreted by spleen cells from immunized mice wither by re-stimulation of the MBP_83-99_ peptide or MBP_83-99_ peptide plus ST285 (Figure 1B). 

### 3.2. ST285 Decreases Secretion of IL-1β, IL-2, and IL-6 by Mouse Spleen Cells

**Figure 2:**
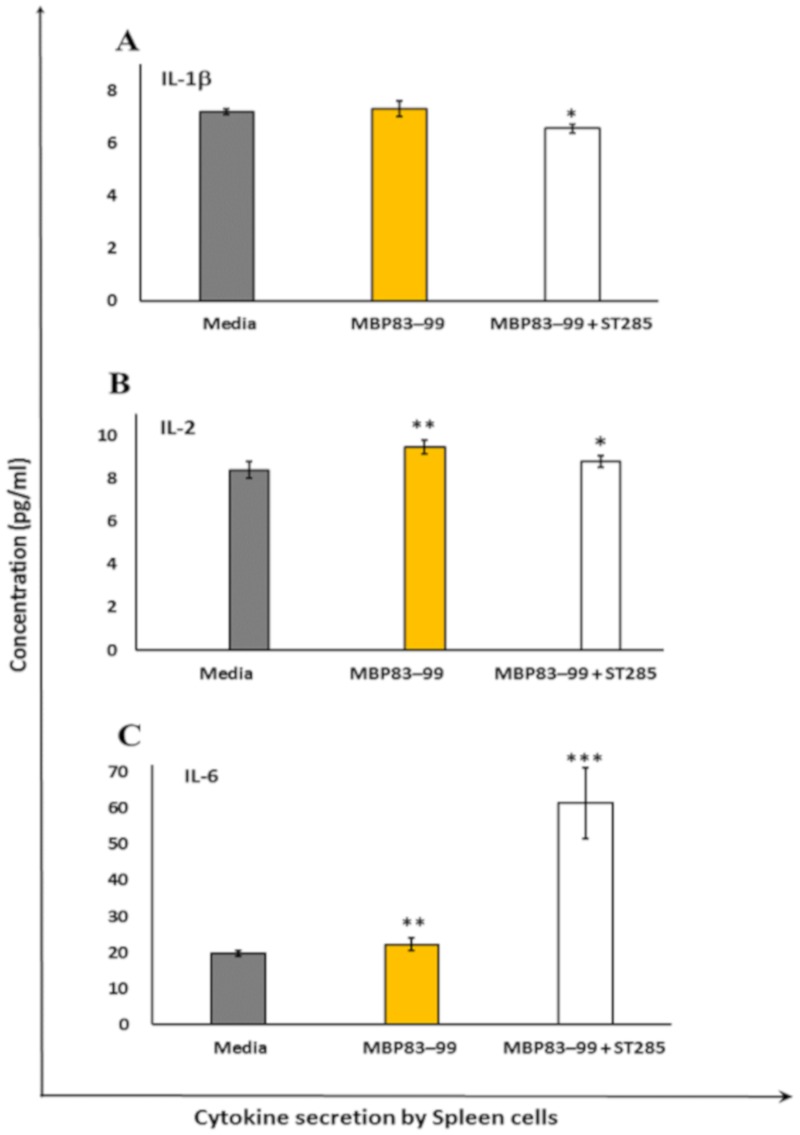
*S. thermophilus* 285 decreases expression of IL-1β, IL-2, and increases IL-6 by mouse spleen cells. Spleen cells isolated from immunized mice (*n* = 3) were stimulated with *S. thermophilus* (ST) ST285 and recall agonist MBP_83–99_ peptide for 24 h and secretion of (**A**) IL-1β, (**B**) IL-2, and (**C**) IL-6 were measured. Recall MBP_83–99_ peptide was used as the reference peptide, and media refers to spleen cells from immunized mice (*n* = 3) without any additional recall peptide or ST285 probiotic bacteria plus MBP_83–99_ peptide. Means are shown as plus or minus (±) standard error of the means. Symbols represent the p value for the Tukey test (one way ANOVA) where * *p* < 0.05 and ** *p* < 0.01 and *** *p* < 0.001.

Secretion of IL-1β was slightly, but significantly reduced in immunized mouse spleen cells re-stimulated with the MBP_83–99_ peptide and ST285 compared to no re-stimulation, or the MBP_83–99_ peptide re-stimulation without ST285 (*p* < 0.05) (Figure 2A). IL-2 production was significantly increased in immunized spleen cells re-stimulated with the MBP_83–99_ peptide (*p* < 0.01), which was weakly but significantly decreased as a result of the co-stimulation of mouse spleen cells with the MBP_83–99_ peptide plus ST285 (*p* < 0.05) (Figure 2B). The production of IL-6 was profoundly increased by immunized mouse spleen cells upon co-culture of ST285 and the recall MBP_83–99_ peptide compared to the control media or MBP_83–99_ recall peptide (*p* < 0.001) (Figure 2C); spleen cells recalled with the MBP_83–99_ peptide alone also increased IL-6 secretion.

### 3.3. ST285 Induces Anti-Inflammatory Cytokine Profile by Mouse Splenocytes

**Figure 3:**
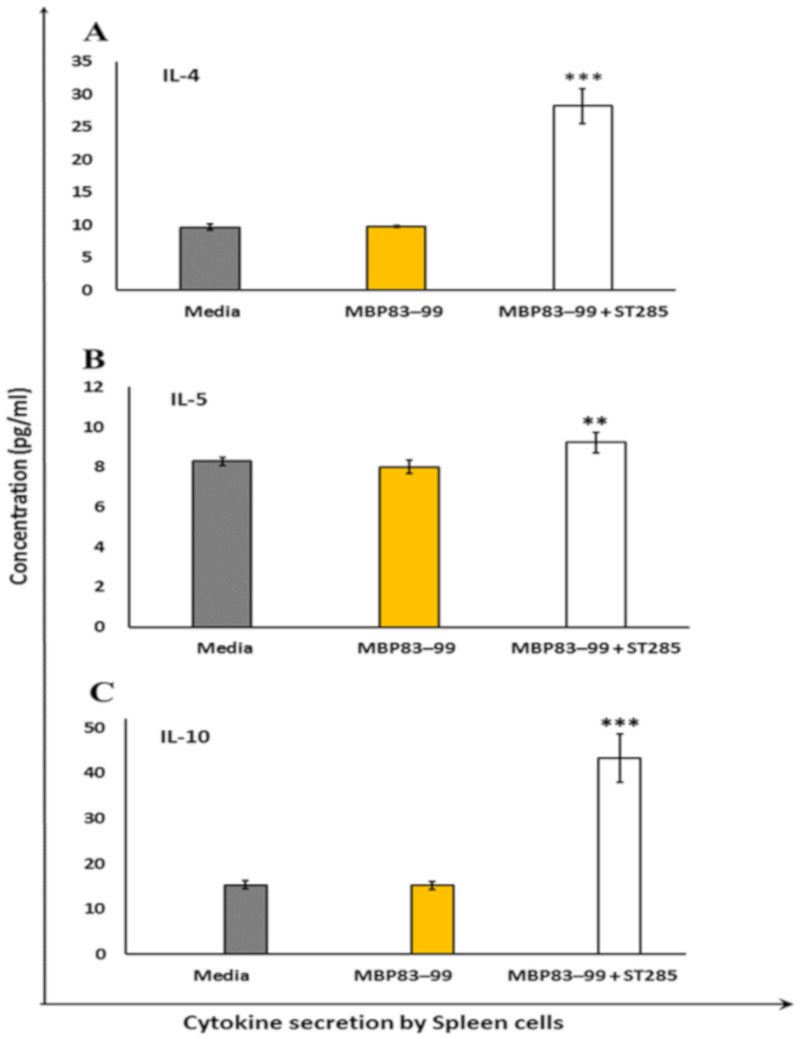
*S. thermophilus* 285 induces the anti-inflammatory cytokine profile by immunized mouse splenocytes. Spleen cells isolated from immunized mice (*n* = 3) were stimulated with *S. thermophilus* (ST) ST285 and the recall agonist MBP_83–99_ peptide for 24 h and the secretion of (**A**) IL-4, (**B**) IL-5, and (**C**) IL-10 were measured. Recall MBP_83–99_ peptide, media alone, or recall MBP_83–99_ peptide plus ST285 are shown from immunized mice (*n* = 3). The means of readings for *n* = 3 mice were calculated and presented as plus or minus (±) the standard error of the mean. Symbols represent the p value for the Tukey test (one way ANOVA) where ** *p* < 0.01 and *** *p* < 0.001.

Mice immunized with the MBP_83–99_ agonist peptide did not induce IL-4, IL-5, and IL-10 anti-inflammatory cytokines in the control (media alone) and recall agonist peptide MBP_83–99_ (Figure 3). However, the Th2 anti-inflammatory cytokine IL-4 was significantly (*p* < 0.001) increased by immunized mouse spleen cells when the MBP_83–99_ recall peptide was co-cultured with ST285 probiotic bacteria (Figure 3A). IL-5 was also increased by immunized spleen cells following co-culture with ST285 and the recall agonist MBP_83–99_ peptide (Figure 3B) (*p* < 0.01). The anti-inflammatory IL-10 cytokine was also significantly increased by immunized mouse spleen cells when co-cultured with ST285 and agonist recall MBP_83–99_ peptide compared to the MBP_83–99_ peptide alone or media control (*p* < 0.001) (Figure 3C).

### 3.4. ST285 Does Not Alter the Secretion of Granulocyte-macrophage Colony-stimulating Factor by Mouse Spleen Cells

**Figure 4:**
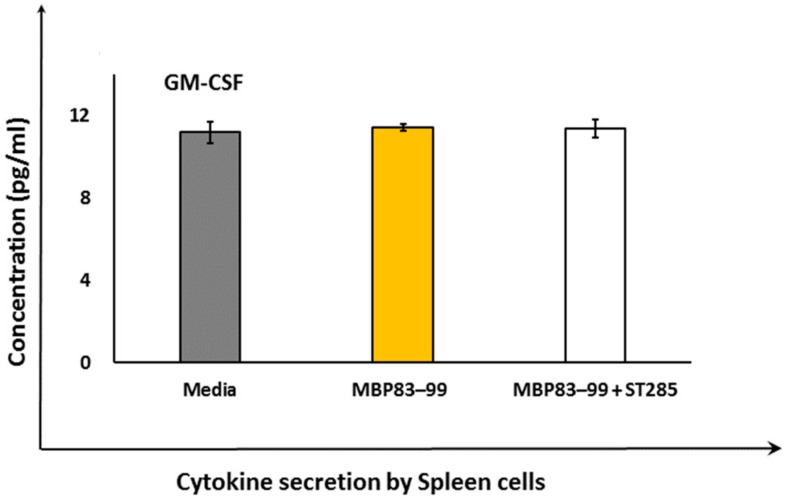
*S. thermophilus* 285 does not alter secretion of GM-CSF by mouse spleen cells. Spleen cells isolated from immunized mice (*n* = 3) were stimulated with *S. thermophilus* (ST) ST285 and the recall reference peptide for 24 hours and secretion of GM-CSF was measured. Recall MBP_83–99_ peptide, media alone, or recall MBP_83–99_ peptide plus ST285 are shown from immunized mice (*n* = 3). The means of readings for *n* = 3 mice were calculated and presented as plus or minus (±) the standard error of the mean.

Secretion of granulocyte-macrophage colony-stimulating factor (GM-CSF) did not show any change by immunized mouse spleen cells upon co-culture with ST285 and agonist recall MBP_83–99_ peptide compared to the negative control or MBP_83–99_ peptide (Figure 4), despite significant upregulation of GM-CSF by ST285 on monocytes/macrophage cells [29].

## 4. Discussion

The Th1 pro-inflammatory cytokines IFN-γ and TNF-α are both involved in the defense against bacterial infections and in acute phase reactions. In MS, these two cytokines are implicated in the pathogenesis of disease by stimulating CD4^+^ T cells against the myelin sheath. Mice immunized with the mannan MBP_83–99_ peptide stimulated IFN-γ secretion, which was reduced in the presence of ST285. This reduction is very important in the context of inflamed situations such as autoimmune and inflammatory diseases, as any reduction in the amount of mediators that cause inflammation is imperative in the relief of symptoms. We previously noted that high levels of TNF-α and IFN-γ was secreted by the U937 monocytic cell line in the presence of ST285 [29]. However, the addition of ST285 to the MBP_83–99_ recall peptide reduced IFN-γ secretion by mouse splenocytes. Spleen cells were populated with B, T, NK cells, macrophages, and monocytes, while the U937 cell line that we previously used were purely monocytic/macrophage cells. Additionally, the polarized inflammatory state of cytokines as a result of the immunization regimen and further exposure of spleen cells to the recall MBP_83–99_ peptide that operate as inflammatory stimuli, compared to the U937 monoclonal cells only being exposed to ST285 bacteria, might give a clue as to the ability of ST285 probiotics to dampen the inflammatory immune response in the instance of exposure to polyclonal spleen cells. 

Secretion of IL-1β by monocytes is involved in regulating the immune and inflammatory responses to infections and injuries; therefore, it has a role in innate immunity. IL-1β is also a major mediator in inflammatory responses associated with various cellular activities such as differentiation, proliferation, and apoptosis [39]. In addition, IL-1β is a regulator of inflammatory reactions and is involved in the stimulation of the central nervous system through cyclooxygenase-2 (PTGS2/COX2), which is involved in neurodegenerative disorders such as MS [33,40], Down’s Syndrome, Alzheimer’s disease, and HIV-associated dementia [41,42].

We noted the secretion of IL-1β by immunized mouse spleen cells was marginally, but significantly reduced in the presence of ST285 with the recall MBP_83–99_ peptide. We previously noted that ST285 did not induce IL-1β cytokine to the U937 cell lines, however, significant upregulation of IL-1β mRNA was induced by human PBMC [31] and monocytes post co-culture with ST285 [32]. It is therefore clear that the immunized mouse spleens and the recall of T cells with the MBP_83–99_ peptide in the presence of ST285 caused a reduction in IL-1β secretion. Likewise, IL-2 was marginally decreased in the presence of ST285 compared to the increased secretion caused by the MBP_83–99_ peptide in the positive control. Co-culturing human PBMC with ST285 also downregulates IL-2 mRNA expression [31].

IL-6 is produced by activated immune cells including DC, B cells, and macrophages. Although IL-6 is associated with acute phase responses, it is also associated with a reduction of Th1 polarization, while promoting Th2 differentiation, B cell maturation, and macrophage differentiation. Proliferation and differentiation of Th2 cells changed the polarized Th1 environment and skewed the Th1/Th2 balance toward Th2, which is beneficial in relieving autoimmune conditions such as MS. IL-6 production was significantly higher (three times) in mouse splenocytes cultured with ST285 compared to the control, hence, it is likely that ST285 bacteria may potentially change the balance toward a healthier state in MS. We previously noted significant upregulation of IL-6 to human monocytes [manuscript submitted] and to bulk PBMC co-cultures [31] with ST285, which are also in accord with the increase in IL-6 levels by the U937 promonocytic cell line co-cultured with ST285 [29]. Likewise, the commercially used probiotic *L. paracasei* DG induces IL-6 cytokines to the THP-1 human monocyte cell line [43]. In contrast, ingestion of *B. bifidum* by mice did not increase the IL-6 levels, but boosted anti-oxidation activities in the spleen and thymus of mice and improved other immune functions by changing the gene expression of immune mediators [44].

It is likely that the constant-shifting in the equilibrium and the dynamics that exist between pro- and anti-inflammatory cytokines will lead to some controversy in the research findings regarding IL-6. On one hand, IL-6 may ease the autoimmune condition due to its downstream immunological effects. On the other hand, elevated levels of pro-inflammatory effector T cell cytokines such as IFN-γ, IL-17 as well as IL-6 are noted in patients with autoimmune myasthenia gravis and MS [45]. Thus, it might be likely that the role that cytokines such as IL-6 play may depend on their bio-environment and may be advantageous to the body, if probiotics such as ST285 are used for neutralization and/or reversing from a pro- to an anti-inflammatory state in the body.

IL-4 is one of the important cytokines required for anti-inflammatory responses against inflammatory conditions such as MS and allergies [29]. IL-4 production was significantly increased by mouse spleen cells in the presence of the recall MBP_83–99_ peptide and ST285 compared to either the MBP_83–99_ peptide alone or the negative control (media). Likewise, it was previously noted that ST285 induced U937 monocytic cells to produce IL-4, although no changes to mRNA expression levels of IL-4 were noted to human monocytes or to bulk human PBMC following co-cultures with ST285 [31]. In contrast, feeding BALB/c mice with *L. paracasei* BEJ01 alone or combined with aflatoxins B1 (AFB1) and fumonisin B1 (FB1) (known foodborne mycotoxins with immunomycotoxic effects on human health) was used to evaluate *L. paracasei* BEJ01 detoxification [46]. Assessing different splenic immunological factors indicated that exposure to these mycotoxins led to increased IL-4 mRNA levels, oxidative stress, and immunotoxicity in the spleen [46] whereas the combined LAB treatment with AFB1 or FB1 suppressed and normalized mRNA levels of IL-4, showing protective effects induced by LAB against AFB1 and FB1 via diminishing toxin adhesion and bioavailability [46]. In contrast, spleen cells isolated from BALB/c mice in vitro co-cultured individually with LAB strains (*L. casei* Lc2w (Lc), *L. plantarum* CCFM47 (Lp), and *L. acidophilus* CCFM137 (La)) showed reduced IL-4 production by spleen cells exposed to La only, while parallel animal studies displayed LAB-induced alleviation of inflammation post airway allergy for all strains through increased Treg cells and modulation of Th1/Th2 balance [47].

The anti-inflammatory cytokine IL-5 is produced by Th2 cells and mast cells. In the event of infection with helminth parasites, IL-5 leads to a lesser risk of autoimmune disorders, which is indirectly accredited to some therapeutic characteristics of IL-5 in autoimmune disorders. We noted a slight increase in the IL-5 production by spleen cells in response to ST285, whereas no changes to the mRNA expression levels of IL-5 were previously noted in ST285 co-cultures with human PBMC or human monocyte cells [manuscripts submitted]. A study showed that treating mice with *Fasciola hepatica* excretion/secretions (FHES) reduced EAE clinical signs due to a significant decrease in the infiltration of Th1 and Th17 cells into the brain and an increase in IL-5 (and IL-23) response, with subsequent increase in eosinophils [48]. It is likely that the small but significant increase of IL-5 may be beneficial to MS. 

IL-10, an anti-inflammatory cytokine, is secreted by Th2 and Treg cells. Amongst all the anti-inflammatory cytokines and chemokines, anti-inflammatory properties of IL-10 are the most potent in suppressing inflammatory mediators by other activated immune cells (TNF-α, IFN-γ, IL-1, IL-17, and IL-23 cytokines) [49]. A significant amplification in the IL-10 levels secreted by the spleen cells in the presence of ST285 was noted, which was similarly shown for the U937 monocytic cell line in the presence of ST285 and to human PBMC, although no significant changes were shown in human monocyte cells [manuscripts submitted]. Likewise, oral administration of *L. reuteri* and *L. lactis* strains to mice stimulated the production of anti-inflammatory IL-10 and Treg cells [50,51]. In addition, sub-clinical studies of *L. salivarius* UCC118, *L. lactis* MG1363, and *L. plantarum* WCFS1 administered to mice and re-exposure of their isolated bone marrow cells to the three bacterial co-cultures showed all three strains differentially stimulated IL-10 production [52]. Correspondingly, when DC from spleen and mesenteric lymph nodes of mice were matured using *L. acidophilus* X37 and exposed to commensal gut *Bifidobacterium longum* Q46, *L. acidophilus* X37, and *Escherichia coli* Nissle 1917, increased IL-10 levels were noted [53]. Similarly, after BALB/c mice were fed with *L. paracasei* BEJ01 alone or combined with aflatoxins B1 and fumonisin B1, high IL-10 mRNA levels were induced [46]. In addition, mice fed with kefir-derived *Lactobacillus kefiri* CIDCA 8348 also increased IL-10 gene expression [54]. In the context of MS, the use of ST285 was shown to downregulate Th1 responses and upregulate Th2 responses, something of the utmost importance to patients with MS to alleviate MS symptoms and/or reversal of the disease [31].

## 5. Conclusions

Immunization of SJL/J mice with agonist MBP_83–99_ peptide conjugated to mannan induces Th1 pro-inflammatory IFN-γ responses and no Th2 anti-inflammatory responses when spleen cells are co-cultured in vitro in the presence of the agonist recall MBP_83–99_ peptide. However, stimulation of spleen cells with the recall MBP_83–99_ peptide in the presence of ST285 significantly increased the secretion of IL-4, IL-6, and IL-10, along with mild upregulation in IL-2 and IL-5, suggesting a role for ST285 in the activation of immune response phenotypes toward a predominant anti-inflammatory profile, tolerance, and suppression of inflammation. In addition, ST285, downregulated the secretion of IL-1a and IFN-γ—the immune mediators involved in Th1 type responses—collectively pointing to a shift in immune responses from Th1 to a Th2 phenotype. More importantly, the significant increase of IL-10 could further contribute by the differentiation of naïve CD4^+^ T cells and proliferation of Tregs, which can also drive the immune balance further toward a dominant anti-inflammatory phenotype. Additionally, given the drastic increase of GM-CSF in our previous studies of ST285 co-cultured with the U937 monocytic cell line, human PBMC, and human monocyte cells, and no change to the secretion of GM-CSF in spleen cells with GM-CSF being a major cytokine for proliferation and recruitment of the immune cells, this might indicate a deliberate and purposeful neutralization of GM-CSF by ST285. The effects of ST285 on the immune response could be used as a novel approach in modulating chronic inflammatory and autoimmune conditions such as MS. Further studies should involve the effects of ST285 in mice with EAE or be used to prevent EAE induction, which will pave the way for new modalities for the treatment of MS in human clinical trials.
